# Correction: Dream content and slow waves benefit prey against predator in a video game confrontation

**DOI:** 10.1038/s41598-026-61691-4

**Published:** 2026-07-09

**Authors:** Daniel S. Brandão, Rafael N. B. Scott, Ernesto S. Soares, Natália B. Mota, Sidarta Ribeiro

**Affiliations:** 1 Brain Institute, Federal University of Rio Grande do Norte (UFRN), Natal, Brazil; 2 Bioinformatics Multidisciplinary Environment (BioME), Digital Metropolis Institute, Federal University of Rio Grande do Norte (UFRN), Natal, Brazil; 3 National Institute of Computational Neuroscience (INCT NeuroComp), Natal, Brazil; 4 Coimbra Institute for Biomedical Imaging and Translational Research (CIBIT), University of Coimbra, Coimbra, Portugal; 5 Research Department, Mobile Brain, Rio de Janeiro, Brazil; 6 Institute of Psychiatry (IPUB), Federal University of Rio de Janeiro (UFRJ), Rio de Janeiro, Brazil; 7 Center for Strategic Studies, Oswaldo Cruz Foundation (FIOCRUZ), Rio de Janeiro, Brazil

Correction to: *Scientific Reports* 10.1038/s41598-026-42759-7, published online 13 March 2026

The original version of this Article contained an error in the Abstract, where it was incorrectly implied that 60 people were sampled.

As a result,

“Electroencephalographic (EEG) and electrocardiographic (ECG) signals were simultaneously recorded from 30 human adult pairs while (1) playing a video game round against each other, (2) taking a nap, (3) reporting dreams and/or thoughts, and (4) playing another round.”

now reads:

“Electroencephalographic (EEG) and electrocardiographic (ECG) signals were simultaneously recorded from 30 human adults paired while (1) playing a video game round against each other, (2) taking a nap, (3) reporting dreams and/or thoughts, and (4) playing another round.”

In addition, there was an error in the Materials and methods section under the subheading ‘Statistical analyses’, where it was incorrectly implied that p-values less than 0.05 were considered marginally significant.

As a result,

"For the a priori comparisons, marginally significant results were observed, defined as those with p-value less than 0.1."

now reads:

"For the a priori comparisons, marginally significant results were observed, defined as those with p-value between 0.05 and 0.1."

The original Article has been corrected.

