# Supplementary material for: Dream content and slow waves benefit prey against predator in a video game confrontation
Source: Sci Rep. 2026 Mar 13;16:9331. doi: 10.1038/s41598-026-42759-7 (PMC13000286; doi:10.1038/s41598-026-42759-7)
Supplement: Supplementary file 1 — Supplementary Material 1 [file 41598_2026_42759_MOESM1_ESM.docx]

Supplementary Materials for

**Dream content and slow waves benefit preys against predators in a video game confrontation**

Daniel Brandão et. al.

*Corresponding authors: Email: daniel.brandao@ufrn.br, sidarta.ribeiro@ufrn.br

**The PDF file includes:**

Tables S1 to S3

Figures S1 and S2

Supplementary Text

**Table S1.** A priori comparison between participants in the prey and predator groups.

| **Variable** | **Predator** | **Prey** | **p-value** | **p-sig** |
| --- | --- | --- | --- | --- |
| **Sex (M/F)** | 11/2 | 12/1 | 0.5393 | n.s. |
| **Gaming Experience (0/1)** | 3/10 | 2/11 | 0.6188 | n.s. |
| **FPS Experience (0/1)** | 2/11 | 2/11 | 1.0000 | n.s. |
| **Interest in FPS games (0/1/2)** | 2/5/6 | 2/4/7 | 0.9103 | n.s. |
| **Age** | 23.9231 | 25.3077 | 0.2208 | n.s. |
| **Total sleep time last night (h)** | 6.0769 | 6.1538 | 0.5334 | n.s. |
| **Epworth (Sleepiness)** | 7.6923 | 7.6923 | 1.0000 | n.s. |
| **Sleep quality** | 1.0769 | 1.0000 | 0.7729 | n.s. |
| **Sleep latency** | 2.3846 | 1.4615 | 0.0515 | n.s. |
| **Sleep duration** | 0.3846 | 0.3077 | 0.7909 | n.s. |
| **Sleep efficiency** | 0.5385 | 0.0769 | 0.1355 | n.s. |
| **Sleep disorders** | 0.9231 | 1.0769 | 0.1831 | n.s. |
| **Sleep medication** | 0.0000 | 0.1538 | 0.3560 | n.s. |
| **Daytime dysfunction** | 1.1538 | 1.2308 | 0.9771 | n.s. |
| **Pittsburgh Total** | 6.4615 | 5.3077 | 0.3789 | n.s. |

**Table S2.** A priori comparison between the two samples of study participants.

| **Variable** | **Sample 1** | **Sample 2** | **p-value** | **p-sig** |
| --- | --- | --- | --- | --- |
| **Sex (M/F)** | 15/3 | 8/0 | 0.2196 | n.s. |
| **Gaming Experience (0/1)** | 3/15 | 2/6 | 0.6188 | n.s. |
| **FPS Experience (0/1)** | 3/15 | 1/7 | 0.7858 | n.s. |
| **Interest in FPS games (0/1/2)** | 4/6/8 | 0/3/5 | 0.3385 | n.s. |
| **Age** | 24.7222 | 24.3750 | 0.7349 | n.s. |
| **Total sleep time last night (h)** | 6.6944 | 4.8125 | 0.1154 | n.s. |
| **Epworth (Sleepiness)** | 7.9444 | 7.1250 | 0.5200 | n.s. |
| **Sleep quality** | 1.0000 | 1.1250 | 0.6845 | n.s. |
| **Sleep latency** | 1.6111 | 2.6250 | 0.1204 | n.s. |
| **Sleep duration** | 0.3889 | 0.2500 | 0.8575 | n.s. |
| **Sleep efficiency** | 0.3333 | 0.2500 | 0.7772 | n.s. |
| **Sleep disorders** | 1.0000 | 1.0000 | 1.0000 | n.s. |
| **Sleep medication** | 0.1111 | 0.0000 | 0.5597 | n.s. |
| **Daytime dysfunction** | 1.1667 | 1.2500 | 0.9010 | n.s. |
| **Pittsburgh Total** | 5.6111 | 6.5000 | 0.3848 | n.s. |

**Table S3.** Partial correlations between score gains and the significant results for dream properties, EEG power and slow wave properties, without covariates (None) and covarying for the score at round 1 (R1), for the previous game experience of the participant (Exp) and for both.

| **Result type** | **Parameter** | **Role** | **N** | **Rho None** | **P value None** | **Rho R1** | **P value R1** | **Rho Exp** | **P value Exp** | **Rho R1 Exp** | **P value R1 Exp** |
| --- | --- | --- | --- | --- | --- | --- | --- | --- | --- | --- | --- |
| **Dream properties** | About game | Prey | 10 | 0.8624 | **0.0013 *** | 0.8974 | **0.0010 *** | 0.8482 | **0.0038 *** | 0.8678 | **0.0052 *** |
| **EEG Power** | Delta (FC6) | Prey | 13 | 0.7785 | **0.0017 *** | 0.8175 | **0.0011 *** | 0.7611 | **0.0040 *** | 0.8144 | **0.0022 *** |
| **EEG Power** | Delta (Cz) | Prey | 13 | 0.7840 | **0.0015 *** | 0.7827 | **0.0026 *** | 0.7563 | **0.0044 *** | 0.7581 | **0.0068 *** |
| **EEG Power** | Delta (CP1) | Prey | 13 | 0.7730 | **0.0019 *** | 0.7771 | **0.0029 *** | 0.7529 | **0.0047 *** | 0.7641 | **0.0061 *** |
| **EEG Power** | Delta (P8) | Prey | 13 | 0.8060 | **0.0008 *** | 0.8485 | **0.0004 *** | 0.7985 | **0.0018 *** | 0.8581 | **0.0007 *** |
| **EEG Power** | Delta (F1) | Prey | 13 | 0.7977 | **0.0010 *** | 0.8191 | **0.0011 *** | 0.7730 | **0.0031 *** | 0.7993 | **0.0031 *** |
| **EEG Power** | Delta (F6) | Prey | 13 | 0.7977 | **0.0010 *** | 0.816 | **0.0012 *** | 0.7755 | **0.0030 *** | 0.8016 | **0.0030 *** |
| **EEG Power** | Delta (FC4) | Prey | 13 | 0.7895 | **0.0013 *** | 0.8013 | **0.0017 *** | 0.7629 | **0.0038 *** | 0.7801 | **0.0046 *** |
| **EEG Power** | Delta (C1) | Prey | 13 | 0.7785 | **0.0017 *** | 0.7752 | **0.0030 *** | 0.7508 | **0.0048 *** | 0.7506 | **0.0077 *** |
| **EEG Power** | Delta (C2) | Prey | 13 | 0.8335 | **0.0003 *** | 0.8516 | **0.0004 *** | 0.8135 | **0.0012 *** | 0.8372 | **0.0013 *** |
| **EEG Power** | Beta (C4) | Prey | 13 | -0.8005 | **0.0010 *** | -0.8136 | **0.0012 *** | -0.7817 | **0.0026 *** | -0.7951 | **0.0034 *** |
| **Slow wave properties** | Summation (FC1) | Prey | 13 | 0.8335 | **0.0003 *** | 0.8391 | **0.0006 *** | 0.8137 | **0.0012 *** | 0.8218 | **0.0019 *** |
| **Slow wave properties** | Summation (F1) | Prey | 13 | 0.7675 | **0.0021 *** | 0.7955 | **0.0019 *** | 0.7393 | **0.0059 *** | 0.7764 | **0.0049 *** |
| **Slow wave properties** | Summation (F6) | Prey | 13 | 0.7730 | **0.0019 *** | 0.8031 | **0.0016 *** | 0.7509 | **0.0048 *** | 0.7925 | **0.0036 *** |

**Table S4.** Correlations between score gains and EEG spectral power during the nap. For every combination of frequency band and role, the results for the channel with smallest p-value is being reported, including all channels with significant correlations. The evaluated frequencies were Delta1 (0.5 to 2.5 Hz), Delta2 (2.5 to 4.5 Hz), Theta (4.5 to 7.5 Hz), Alpha (7.5 to 12.5 Hz), Sigma (12.5 to 16.5 Hz) and Beta (14.5 to 30.5 Hz).

| **Role** | **Frequency band** | **Channel** | **N** | **Rho** | **Adjusted p value** |
| --- | --- | --- | --- | --- | --- |
| **Predator** | Delta1 | P4 | 13 | -0.6510 | 0.1769 |
| **Predator** | Delta2 | C6 | 13 | -0.4855 | 0.7799 |
| **Predator** | Theta | PO8 | 13 | 0.44130 | 0.9202 |
| **Predator** | Alpha | FC1 | 13 | -0.2951 | 0.8989 |
| **Predator** | Sigma | CP2 | 13 | 0.2731 | 0.9979 |
| **Prey** | **Delta1** | **FC1** | **13** | **0.7840** | **0.0362 *** |
| **Prey** | **Delta1** | **FC6** | **13** | **0.7895** | **0.0333 *** |
| **Prey** | **Delta1** | **Cz** | **13** | **0.7647** | **0.0478 *** |
| **Prey** | **Delta1** | **CP1** | **13** | **0.7675** | **0.0458 *** |
| **Prey** | **Delta1** | **P8** | **13** | **0.7922** | **0.0320 *** |
| **Prey** | **Delta1** | **F1** | **13** | **0.7702** | **0.0441 *** |
| **Prey** | **Delta1** | **F6** | **13** | **0.7620** | **0.0496 *** |
| **Prey** | **Delta1** | **C2** | **13** | **0.7702** | **0.0441 *** |
| **Prey** | **Delta2** | **FC6** | **13** | **0.8115** | **0.0287 *** |
| **Prey** | **Delta2** | **P8** | **13** | **0.8005** | **0.0356 *** |
| **Prey** | **Delta2** | **C2** | **13** | **0.7867** | **0.0449 *** |
| **Prey** | Theta | FT7 | 13 | 0.6602 | 0.2392 |
| **Prey** | Alpha | C4 | 13 | -0.4951 | 0.4206 |
| **Prey** | Sigma | FT7 | 13 | 0.5997 | 0.4803 |
| **Prey** | **Beta** | **C4** | **13** | **-0.8005** | **0.0424 *** |

**Fig. S1. No significant correlations were found between score gains and spindle properties.** (**A**) Topographies of Spearman correlation coefficients (Rho) between score gains and spindle properties. (**B**) Scatter plot showing the correlation between score gain and the spindle duration at channel FC6 during sleep.


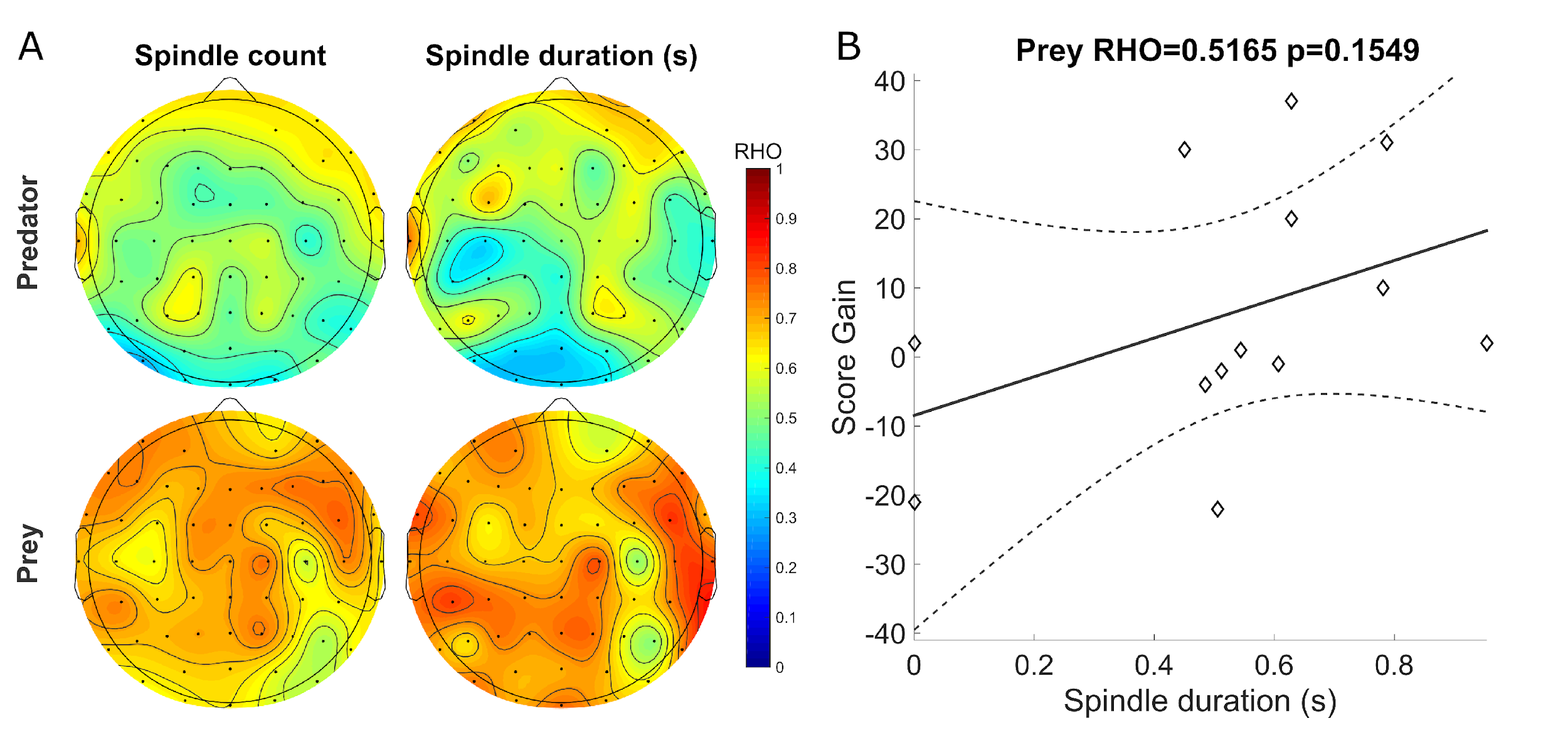


**Figure S2.** Comparison of duration and percentage for each sleep stage between prey and predator roles.


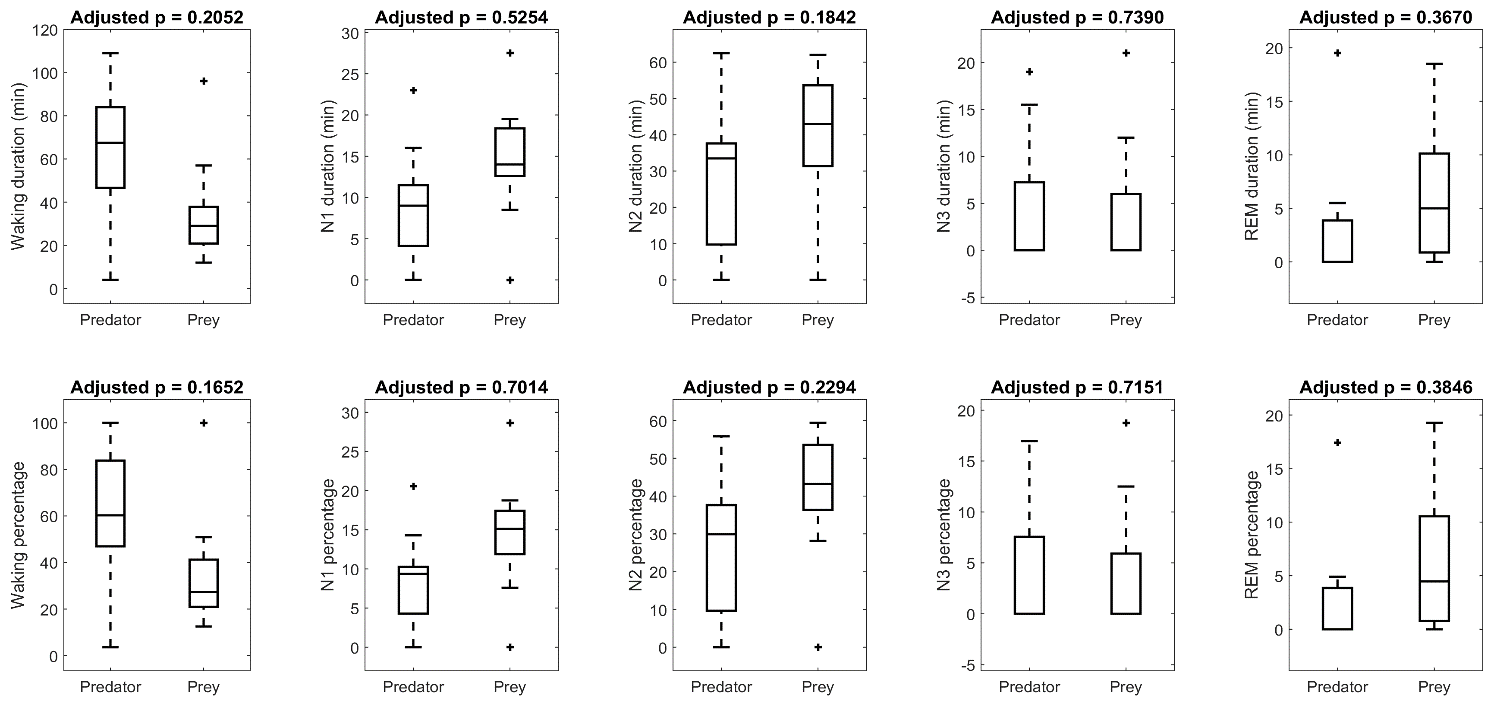


**Supplementary Text**

Representative example of a dream report highly related to the game (rating = 4.5)

Well, I dreamed about several things. First, what I remember is that I actually dreamt about the experiment I was doing here. I dreamt that the researcher was talking to me about the experiment and that there were other types of chairs here in the experiment. I remember there were some chairs here. Then I dreamt that my girlfriend was here too, and she stayed here during the experiment, watching how me and the other player were doing. Afterwards I dreamt about the game. I dreamed about playing the game again.

Representative example of a dream report highly unrelated to the game (rating = 1.5)

I was dreaming that I was on a beach. There were a lot of people and I was with my dog until it rained and then I started dreaming about my grandparents
